# Incidence of *Treponema pallidum* in donors from a blood centre in Colombia, 2012–2024

**DOI:** 10.1017/S0950268826101721

**Published:** 2026-06-04

**Authors:** Lucrecia González-Tamayo, Sara Saldarriaga-Vélez, Juliana Villa-Carrasquilla, Alejandro Gil-Betancur, Jaiberth Antonio Cardona-Arias

**Affiliations:** University of Antioquia, Colombia

**Keywords:** blood donors, incidence, *Treponema pallidum*

## Abstract

Syphilis remains a significant transfusion-transmitted infection. In Colombia, routine epidemiological surveillance primarily targets pregnant women, leaving the burden of infection among the apparently healthy population. This study determined the incidence of *Treponema pallidum* and its associated factors in donors at a blood centre in Colombia between 2012 and 2024. A retrospective cohort study was conducted, analyzing 64,166 repeat blood donors. Incidence was calculated using 95% confidence intervals. Associations with demographic and donation-related variables were assessed using Chi-square test and potential confounders were adjusted using a multivariable regression model. The overall incidence of syphilis was 5.1 per 1,000 donors. The associated factors included age, sex, occupation, collection site, and donor type. Higher incidence proportions were observed in male donors (*RR* = 1.76), individuals aged between 60 and 65 years (*RR* = 2.43), unemployed individuals (*RR* = 3), donors collected at the centre (*RR* = 1.45), and replacement donors (*RR* = 3.51). These incidences indicate ongoing transmission within a low-risk population. The highest incidence in some groups enabled the generation of hypotheses about differential exposure patterns, guiding subsequent aetiological studies, and optimizing donor selection. Understanding local epidemiology is essential for designing public health interventions tailored to the specific epidemiological profile.

## Introduction

Blood donation is a procedure in which all or some blood components are collected for subsequent transfusion to patients with blood loss or low blood cell counts [1, 2]. This treatment can cause adverse reactions, such as transfusion-transmitted infections, which account for approximately 40% of adverse events [3, 4]. Consequently, the safety of transfusion depends primarily on rigorous donor selection processes, including standardized medical interviews, self-deferral strategies, and highly sensitive laboratory screening for mandatory serological markers [3, 4]. Despite these measures, certain infections continue to challenge the safety of blood. Syphilis remains a concern due to its prolonged latency, heterogeneous clinical presentation, and potential for serological under detection at specific stages of infection, which may compromise transfusion safety if not adequately identified [5, 6].

Syphilis is an infectious disease caused *by Treponema (T.) pallidum.* Based on its clinical presentation, infectivity, and progression, it can be classified into different stages, with manifestations ranging from painless papules or pustules to cardiovascular and nervous systems involvement [6]. The primary route of transmission is sexual contact; the bacterium penetrates the mucous membranes of the genitals or the skin, infiltrating the lymphatic and blood systems [7]. Vertical transmission is also possible [7], particularly during the early stages of infection, leading to congenital syphilis, which can result in miscarriage, perinatal death, or multiple malformations and deficiencies (auditory, neurological, growth) in the foetus [8]. Blood transfusions represent another mode of transmission, with most reported cases occurring when the disease is in the primary or secondary stage. *T. pallidum* blood concentrations vary and tend to be short-lived, even after recent infection [9].

Global epidemiological data indicate a sustained increase in the incidence syphilis over recent decades. A retrospective analysis conducted between 1990 and 2017 reported a 10.6% increase in global incidence from 119.54 to 132.26 cases per 100,000 population [10]. More recently, the Pan American Health Organization reported a 30% increase in new syphilis cases among adults aged 15–49 years between 2020 and 2022, highlighting a renewed public health concern in the region [11]. The epidemiology of syphilis in blood donor populations shows distinct patterns. Studies from Israel reported an incidence of 8 per 100,000 donors between 2005 and 2009, representing an 11.4-fold increase compared with previous reports [12]. In the United States, an incidence proportion of 10.8 per 100,000 people per year was reported between 2020 and 2022, with a higher incidence in men, African Americans, and young people aged 18–38 years [13].

In Colombia, studies conducted in different regions have reported a heterogeneous and increased prevalence of syphilis among blood donors. In Barranquilla, the prevalence was 0.93% from 2015 to 2016, whereas in Antioquia, specifically in Medellín, the prevalence among donors was 0.6% between 2007 and 2010, with a significant increase in subsequent years. A study conducted between 2010 and 2013 reported a prevalence of 1.3%, more than double that recorded in the previous period [3, 5, 14]. Previous studies have described differential prevalence according to demographic and donation-related characteristics, including age, sex, occupation, donation frequency, type of donation, collection site, and place of residence [3]. However, these patterns differ from those reported in the general population, where risk has been primarily associated with sexual behaviour and structural barriers to healthcare access, diagnosis, and treatment [10, 11].

Despite extensive data on the prevalence of syphilis, information on the incidence of *T. pallidum* infection in Colombia remains limited and outdated, particularly among blood donors. Reliable incidence estimation requires repeated testing of the same individuals over a defined period, a process that is challenging within national surveillance systems. Longitudinal monitoring of repeat donors is essential because it allows for the identification of new infections within a defined time window and provides a more accurate measure of transmission dynamics than prevalence alone [15].

This study aimed to determine the incidence of *T. pallidum* and its associated factors in donors at a blood centre in Colombia between 2012 and 2024.

## Methods

### Study type

A retrospective observational cohort study was conducted with donors who made at least two donations at the Blood Centre in 1 year. The cohort was open, the inclusion was based on a first donation occurring between January 2012 and December 2024, and at least one additional donation was made within the next 12 months.

### Study population

The study included 64,166 repeat donors who attended a blood centre from Antioquia during the study period, with at least one previous donation that tested negative for *T. pallidum.* The blood centre is responsible for collecting, processing, storing, and distributing blood components, including screening for serological markers. Additionally, it participates in a network of blood banks in Antioquia and is recognized as a regional and national benchmark for the quality of its processes. It is one of Colombia’s largest blood centres. All eligible donors were included in the study; therefore, no sample size calculation or sampling strategy was applied.

All criteria established in the technical guidelines of the National Institute of Health of Colombia for selecting blood donors were applied. This includes pre-donation counselling, review of previous donations in the blood bank databases and the hemovigilance information system, pre-donation self-exclusion based on some cosmetic procedures, use of specific medications and psychoactive drugs, travel to endemic areas, or some health conditions, and risky sexual behaviour that includes people who have changed sexual partners in less than 3 months, had multiple sexual partners in less than 6 months, or had sexual contact with key groups such as prisoners, sex workers, men who have sex with men, and people with a previous STI diagnosis. The selection process was concluded with an interview and physical examination, including weight, blood pressure, and haemoglobin measurements.

### Information collection **and bias control**

Secondary data were obtained from the blood centre’s information system (Hexabank, license 1.28.30.50). Extracted variables included place of residence, age group, occupation, sex, donation or collection site (headquarters or extramural campaign), type of donor classified in altruistic or replacement (donate blood motivated by the need of a specific patient-family member, friend or acquaintance, to substitute the units that the hospital used in their treatment), and *T. pallidum* screening test result.

The screening test used for all serological markers at the blood centre is chemiluminescent microparticle immunoassay, which has a sensitivity of ≥99.0% and a specificity of 99.9% [3]. In Colombian blood banks, positive cases must follow this procedure: repeat the test in duplicate, in a tube and blood bag, with the same sample and diagnostic test; if one or both results are positive, the patient is referred to their healthcare provider for diagnostic confirmation and to determine clinical management.

Biases were controlled through duplicate data extraction. Concerning the quality of the primary data, trained personnel collected information at the bank at all stages of donor selection. Diagnostic tests demonstrate excellent validity, and the bank implements internal and external quality control.

### Data analysis

Sociodemographic characteristics and donation-related variables were summarized using absolute and relative frequencies. The incidence of *T. pallidum* among repeat donors was calculated using 95% confidence interval. Pearson’s chi-square test was used for nominal variables to identify associated factors, and trend tests were applied for ordinal variables. A multivariate log-binomial regression was performed to estimate the adjusted relative risks for each independent variable, while controlling for confounding factors. The dependent variable was the incidence of *T. pallidum*; independent variables were selected using the Hosmer–Lemeshow criterion (*p*-value <0.25 in bivariate analysis), including only those statistically significant in the multivariate adjustment. Analyses were conducted using SPSS version 29.0, and p-values less than 0.05 were considered statistically significant.

### Ethical aspects

This study adhered to the principles outlined in Resolution 8,430 by 1993 of the Colombian Ministry of Health which classifies the research as ‘risk-free’ [16]; Decree 1,377 of 2013 [17] and Article 15 of the Political Constitution of Colombia, which protects participants’ personal data [18]. Additionally, Resolution 1995 of 1999 was applied, establishing regulations for the handling of medical records to ensure the protection and confidentiality of personal information [19]. The project was approval by the scientific committee of the Blood Centre, and all donors provided their informed consent.

## Results

During the study period, blood bank registered 86,476 first-time donors, 86,220 non-repeat donors (those who registered more than one donation but over a period of time longer than 1 year), and 64,166 repeat donors (who donated twice in less than a year).

The study population consisted predominantly of men (52.4%), with most of the donors residing in Medellín (60.4%). The majority were aged between 27 and 44 years (80%) and were employed (36.1%). Most donations were altruistic (78.8%) and were collected during blood donation campaigns organized by the Blood Centre (59.7%) (Table 1).

**Table 1.** Sociodemographic description and donor type in the study population Table 1. long description.

Variables and their levels		*N*	*%*
Place of residence	Medellín	38,749	60.4
	Another in the Aburrá Valley	19,733	30.8
	Another in Antioquia	5,485	8.5
	Outside Antioquia	199	0.3
Age group	Young person (18–26)	6,664	10.4
	Young adult (27–44)	35,130	54.7
	Average adult (45–59)	16,233	25.3
	Elderly person (60–65)	6,139	9.6
Sex	Woman	30,547	47.6
	Man	33,619	52.4
Occupation	Unemployed	788	1.2
	Homemaker	4,769	7.4
	Student	13,539	21.1
	Assistant	4,278	6.7
	Employee	23,139	36.1
	Self-employed	5,166	8.1
	Professional	12,487	19.5
Collection	Headquarters	25,866	40.3
	Campaign	38,300	59.7
Donor type	Altruistic	50,593	78.8
	Replacement	13,573	21.2

Among repeat donors, 326 cases of *T. pallidum* infection were identified, resulting in an incidence of 5.1 cases per 1,000 donors. The incidence varied significantly by age group, sex, occupation, collection site, and donor type.

A higher incidence proportion was observed among donors aged 60–65 years (10.9 per 1,000) and men (6.5 per 1,000). An increased proportion was also found among unemployed donors (10.2 per 1,000), those donating at the blood centre (6.61 per 1,000), and replacement donors (10.8 per 1,000) (Table 2).

**Table 2.** Overall incidence of *T. pallidum* and specific incidence according to sociodemographic characteristics and donor type Table 2. long description.

Variables and their levels	*N* positive	Incidence/1.000 (95% CI)	*p*-value Chi^2^
Place of residence			
Medellín	212	5.47 (4.72–6.22)	0.212^a^
Another one from the Aburrá Valley	85	4,31 (3.37–5.25)	
Another in Antioquia	27	4.92 (2.98–6.87)	
Outside Antioquia	2	10.05 (1.22–25.83)	
Age group			
Young person (18–26)	22	3.30 (1.85–4.75)	<0.001*^b^
Young adult (27–44)	134	3.81 (3.16–4.47)	
Average adult (45–59)	103	6.35 (5.09–7.60)	
Elderly person (60–65)	67	10.91 (8.23–13.59)	
Sex			
Woman	108	3.54 (2.85–4.22)	<0.001* ^a^
Man	218	6.48 (5.61–7.36)	
Occupation			
Unemployed	8	10.15 (2.52–17.79)	<0.001*^a^
Housewife	29	6.08 (3.77–8.39)	
Student	49	3.62 (2.57–4.67)	
Assistant	19	4.44 (2.33–6.55)	
Formal or informal employee	159	6.87 (5.79–7.96)	
Independent	27	5.23 (3.16–7.29)	
Professional	35	2.80 (1.84–3.77)	
Collection			
Headquarters	171	6.61 (5.60–7.62)	<0.001*^a^
Campaign	155	4.05 (3.40–4.70)	
Donor type			
Altruistic	180	3.56 (3.03–4.09)	<0.001*^a^
Replacement	146	10.76 (8.98–12.53)	

a Pearson’s chi-square.

b Trend chi-square.

* *p*-values less than 0.001 are reported as *p* < 0.001.

After adjusting for potential confounders, statistically significant differences in the incidence of *T. pallidum* infection were identified based on age group, occupation, place of recruitment, and donor type. Donors aged 60–65 years had a 2.4-fold higher incidence compared with those aged 18–27 years. The incidence was three times higher among unemployed donors than among professionals; 45% higher in donations collected at the Blood Centre headquarters compared to campaign-based collections; and 3.5 times higher in replacement donors compared to altruistic donors. No significant differences were observed between donors aged 27–59 years and the reference group (18–26 years), or between self-employed workers and employees (*p* > 0.05) (Table 3).

**Table 3.** Multivariate regression model for factors associated with the incidence of *T. pallidum* Table 3. long description.

Variables included in the model	*β*	*p*	RR (CI 95%)
Age group		0.000	
27–44/18–26	0.011	0.964	1.01 (0.62–1.64)
45–59/18–26	0.390	0.153	1.48 (0.86–2.52)
60–65/18–26	0.889	0.002	2.43 (1.39–4.26)**
Gender (Male/Female)	0.568	0.000	1.76 (1.36–2.30)**
Occupation		0.010	
Unemployed/Professional	1.092	0.006	3.00 (1.37–6.47)**
Housewife/Professional	0.736	0.007	2.09 (1.22–3.57)**
Estudiante/Professional	0.560	0.020	1.75 (1.09–2.80)*
Assistant/Professional	0.561	0.049	1.75 (1.00–3.07)*
Employee/Professional	0.656	0.001	1.93 (1.33–2.79)**
Independent/Professional	0.310	0.231	1.36 (0.82–2.26)
Collection (Headquarters/Campaign)	0.369	0.028	1.45 (1.04–2.01)*
Type of donor (Replacement/Altruistic)	1.254	<0.001	3.51 (2.52–4.88)**

* *p* < 0.05.

** *p* < 0.01.

## Discussion

The sociodemographic profile of donors in this study was broadly consistent with previous reports from other regions of the country, where blood donors are predominantly men, employed individuals, and young adults [5, 20, 21]. However, studies of donors in Medellín have reported a higher proportion of female donors and altruistic donations [14, 17], while others have noted a predominance of students under 30 years of age [3]. Contextual factors, including local recruitment strategies and donor deferral practices, likely influence these variations. Although women generally exhibit a lower prevalence of infections, higher deferral rates due to lower haemoglobin levels may reduce the representation among eligible donors [22]. Similarly, the overrepresentation of young donors in some studies may be attributable to targeted donation campaigns in academic and occupational settings, which preferentially engage younger populations [3, 20].

Incidence proportion was 5.1 cases per 1,000 donors, which coincides with global trends showing an increase in the incidence of syphilis [23]; as well as reports from specific countries such as India where figures between 0.01% and 0.77% have been reported in women in antenatal clinic attendees [24], and specific studies with blood donors in whom the rising incidence of syphilis over a 12-year period has been reported, this constitutes a serious public health alert because it involves healthy and symptom-free people [25].

A higher incidence was observed among male donors. This sex-based difference aligns with findings from other studies, although there is considerable variability in incidence estimates. For example, a study conducted in the United States reported an incidence of 1.4 per 1,000 donations, which is substantially lower than the 6.5 per 1,000 observed in the present study [13]. Similarly, data from France showed a higher incidence among male donors, ranging from 2.2- to 4.1-fold compared with female donors during certain years of follow-up. This increase was attributed to a growing proportion of male donors reporting sex with other men on time, with a prevalence of 16.7% in 2007 and 64.9% in 2022 [26]. It is important to consider that some men who have sex with men could register a higher risk of infection; according to some authors they report less frequent or inconsistent condom use and a higher number of sexual partners [27]. These factors may contribute to the observed sex differences in incidence and underscore the need for continuous evaluation of donor selection strategies and epidemiological surveillance to ensure transfusion safety.

A similar pattern was observed in the age-stratified analyses, which consistently demonstrated an association between *T. pallidum* seroreactivity and age, although the incidence varied substantially across age groups. In contrast to a previous study that reported the highest incidence among older adults aged 60–65 years (10.9 per 1,000 donors) [13], this higher incidence in the older group may be studied with a combination of behavioural, structural, and generational factors. These include limited exposure to sexual health education programmes historically targeted towards younger populations, lower perceived risk of sexually transmitted infections, and reduced condom use among older adults [28, 29]. These findings may be partially influenced by characteristics inherent to blood donor populations, including selection criteria and health-seeking behaviour, which may differ across age groups and potentially affect observed incidence patterns.

Given the close relationship between prevalence and incidence and the limited availability of incidence studies in Colombia, the occupation-specific findings observed in this study can be contextualized using seroprevalence data from Medellín, which demonstrate a similar pattern of lower *T. pallidum* seroreactivity among professionals and students [3]. This pattern may reflect differential access to preventive measures, sexual health education, and institutional health promotion initiatives, including information and communication campaigns implemented in educational settings and workplaces. In contrast, unemployed individuals or homemakers may face greater barriers to accessing these preventive resources and health services, potentially increasing their vulnerability to infection [30].

Regarding the variables related to the donation process (collection site and donor type), replacement donors and those recruited at the headquarters (intramural) exhibited a significantly higher risk of *T. pallidum* infection. These findings are consistent with previous reports indicating a lower infection risk among altruistic and campaign-based donors. The elevated risk observed among replacement donors may be partially explained by the contextual and procedural factors inherent to this donation modality. Replacement donors and donors recruited at headquarters are often motivated by the immediate need to support a family member or acquaintance, which may increase the likelihood of incomplete or inaccurate disclosure of risk behaviours during pre-donation screening and self-exclusion processes. This, in turn, compromises donor selection and raises the probability of unsafe blood unit collection [3, 31, 32, 33].

## Limitations

The limitations of this study include its observational design, which does not allow the establishment of causal relationships. Therefore, the results only demonstrate associations between *T. pallidum* seroreactivity and the variables analyzed, and these findings should be interpreted as exploratory. Further studies are required to improve the aetiological design that allows the explanatory mechanisms of the differences in some population subgroups to be expanded. Additionally, the lack of information on relevant behavioural, clinical, and socioeconomic factors – such as sexual practices, prior sexually transmitted infections, and access to healthcare – restricted the ability to comprehensively assess the determinants of donor reactivity. Other potentially associated variables were not considered, which would have provided a broader and more complete understanding of the infection’s behaviour. This demonstrates the problem of residual confusion and limited generalizability. Although the test is 99% sensitive, false negatives on the initial screen are also possible, resulting in the discarding of the blood unit despite the false results can be ruled out in the confirmatory test.

## Conclusion

A high incidence of *T. pallidum* was found among donors, a population typically considered to have no risk factors, demonstrating the ongoing circulation of this pathogen within apparently healthy groups. The highest incidence in some groups enabling the formulation of hypotheses regarding differential exposure patterns, guiding subsequent aetiological studies, and optimizing donor selection. Furthermore, understanding local epidemiological patterns is essential for designing targeted public health interventions tailored to the specific characteristics of the population served.

## Data Availability

The data supporting of this study are available from the corresponding author upon reasonable request.

## Long descriptions

### Table 1. Long description

The table contains four columns: Variables and their levels, N, and percent.

* Place of residence: Medellín has 38,749 (60.4 percent); Another in the Aburrá Valley has 19,733 (30.8 percent); Another in Antioquia has 5,485 (8.5 percent); Outside Antioquia has 199 (0.3 percent).

* Age group: Young person 18 to 26 has 6,664 (10.4 percent); Young adult 27 to 44 has 35,130 (54.7 percent); Average adult 45 to 59 has 16,233 (25.3 percent); Elderly person 60 to 65 has 6,139 (9.6 percent).

* Sex: Woman has 30,547 (47.6 percent); Man has 33,619 (52.4 percent).

* Occupation: Unemployed has 788 (1.2 percent); Homemaker has 4,769 (7.4 percent); Student has 13,539 (21.1 percent); Assistant has 4,278 (6.7 percent); Employee has 23,139 (36.1 percent); Self-employed has 5,166 (8.1 percent); Professional has 12,487 (19.5 percent).

* Collection: Headquarters has 25,866 (40.3 percent); Campaign has 38,300 (59.7 percent).

* Donor type: Altruistic has 50,593 (78.8 percent); Replacement has 13,573 (21.2 percent).

Navigate back to Table 1..

### Table 2. Long description

The table consists of four columns: Variables and their levels, N positive, Incidence per 1,000 with 95 percent C I, and p-value Chi-squared.

Place of residence:

- Medellín: 212 positive, 5.47 incidence.

- Another one from the Aburrá Valley: 85 positive, 4.31 incidence.

- Another in Antioquia: 27 positive, 4.92 incidence.

- Outside Antioquia: 2 positive, 10.05 incidence.

- p-value: 0.212.

Age group:

- Young person 18 to 26: 22 positive, 3.30 incidence.

- Young adult 27 to 44: 134 positive, 3.81 incidence.

- Average adult 45 to 59: 103 positive, 6.35 incidence.

- Elderly person 60 to 65: 67 positive, 10.91 incidence.

- p-value: less than 0.001.

Sex:

- Woman: 108 positive, 3.54 incidence.

- Man: 218 positive, 6.48 incidence.

- p-value: less than 0.001.

Occupation:

- Unemployed: 8 positive, 10.15 incidence.

- Housewife: 29 positive, 6.08 incidence.

- Student: 49 positive, 3.62 incidence.

- Assistant: 19 positive, 4.44 incidence.

- Formal or informal employee: 159 positive, 6.87 incidence.

- Independent: 27 positive, 5.23 incidence.

- Professional: 35 positive, 2.80 incidence.

- p-value: less than 0.001.

Collection:

- Headquarters: 171 positive, 6.61 incidence.

- Campaign: 155 positive, 4.05 incidence.

- p-value: less than 0.001.

Donor type:

- Altruistic: 180 positive, 3.56 incidence.

- Replacement: 146 positive, 10.76 incidence.

- p-value: less than 0.001.

Navigate back to Table 2..

### Table 3. Long description

The table consists of four columns: Variables included in the model, beta, p, and R R (C I 95 percent).

* Age group: Overall p-value is 0.000.

- 27 to 44 versus 18 to 26: beta 0.011, p 0.964, R R 1.01 (0.62 to 1.64).

- 45 to 59 versus 18 to 26: beta 0.390, p 0.153, R R 1.48 (0.86 to 2.52).

- 60 to 65 versus 18 to 26: beta 0.889, p 0.002, R R 2.43 (1.39 to 4.26).

* Gender (Male or Female): beta 0.568, p 0.000, R R 1.76 (1.36 to 2.30).

* Occupation: Overall p-value is 0.010.

- Unemployed versus Professional: beta 1.092, p 0.006, R R 3.00 (1.37 to 6.47).

- Housewife versus Professional: beta 0.736, p 0.007, R R 2.09 (1.22 to 3.57).

- Estudiante versus Professional: beta 0.560, p 0.020, R R 1.75 (1.09 to 2.80).

- Assistant versus Professional: beta 0.561, p 0.049, R R 1.75 (1.00 to 3.07).

- Employee versus Professional: beta 0.656, p 0.001, R R 1.93 (1.33 to 2.79).

- Independent versus Professional: beta 0.310, p 0.231, R R 1.36 (0.82 to 2.26).

* Collection (Headquarters or Campaign): beta 0.369, p 0.028, R R 1.45 (1.04 to 2.01).

* Type of donor (Replacement or Altruistic): beta 1.254, p less than 0.001, R R 3.51 (2.52 to 4.88).

Navigate back to Table 3..
